# Measuring the neighbourhood using UK benefits data: a multilevel analysis of mental health status

**DOI:** 10.1186/1471-2458-7-69

**Published:** 2007-05-03

**Authors:** David L Fone, Keith Lloyd, Frank D Dunstan

**Affiliations:** 1 Department of Primary Care & Public Health, Centre for Health Sciences Research, Cardiff University, Heath Park, Cardiff CF14 4YS, UK; 2 School of Medicine, Swansea University, Swansea SA2 8PP, UK

## Abstract

**Background:**

Evidence from multilevel research investigating whether the places where people live influence their mental health remains inconclusive. The objectives of this study are to derive small area-level, or contextual, measures of the local social environment using benefits data from the Department of Work and Pensions (DWP) and to investigate whether (1) the mental health status of individuals is associated with contextual measures of low income, economic inactivity, and disability, after adjusting for personal risk factors for poor mental health, (2) the associations between mental health and context vary significantly between different population sub-groups, and (3) to compare the effect of the contextual benefits measures with the Townsend area deprivation score.

**Methods:**

Data from the Welsh Health Survey 1998 were analysed in Normal response multilevel models of 24,975 individuals aged 17 to 74 years living within 833 wards and 22 unitary authorities in Wales. The mental health outcome measure was the Mental Health Inventory (MHI-5) of the Short Form 36 health status questionnaire. The benefits data available were the means tested Income Support and Income-based Job Seekers Allowance, and the non-means tested Incapacity Benefit, Severe Disablement Allowance, Disability Living Allowance and Attendance Allowance. Indirectly age-standardised census ward ratios were calculated to model as the contextual measures.

**Results:**

Each contextual variable was significantly associated with individual mental health after adjusting for individual risk factors, so that living in a ward with high levels of claimants was associated with worse mental health. The non-means tested benefits that were proxy measures of economic inactivity from permanent sickness or disability showed stronger associations with individual mental health than the means tested benefits and the Townsend score. All contextual effects were significantly stronger in people who were economically inactive and unavailable for work.

**Conclusion:**

This study provides evidence for substantive contextual effects on mental health, and in particular the importance of small-area levels of economic inactivity and disability. DWP benefits data offer a more specific measure of local neighbourhood than generic deprivation indices and offer a starting point to hypothesise possible causal pathways to individual mental health status.

## Background

It is now generally accepted that the places where people live are an important factor in determining and sustaining inequalities in health outcome between individuals [1-3]. Despite the wide spatial variation described in mental health status in Great Britain [4-6], evidence from multilevel modelling studies to support the hypothesis that that where you live is important to individual mental health status is inconsistent, both in Great Britain and internationally. Some studies suggest that small area deprivation at the level of the census ward is associated with individual mental health status [7-11], and this effect might be stronger in people who are economically inactive [8,10]. However other studies from Great Britain, the Netherlands, and the USA have found little evidence for an effect of area deprivation [12-18]. A second neighbourhood exposure of interest as a possible determinant of population health is social capital, defined by Putnam as "features of social organisation, such as trust, norms, and networks, that can improve the efficiency of society by facilitating coordinated actions" [19]. Here again the evidence for an ecological effect of social capital on mental health outcomes is inconclusive [20], although a recent study has suggested that small-area social cohesion may modify associations between individual mental health and area income deprivation [21].

One of the principal challenges in investigating associations between the mental health status of individuals and characteristics of the local area or neighbourhood is measuring the characteristics of the neighbourhood that can be hypothesised to be related to individual mental health. In the absence of agreement on methods to define a neighbourhood in a way that results in a geographically defined area [22-24], multilevel studies of neighbourhoods and health have generally used administrative boundaries, such as the census ward, as the best available proxy. The crucial question is whether characteristics measured for these administrative areas are *a priori *believed to capture homogeneous social and cultural groupings of individuals that may represent 'neighbourhood' and can influence health through some mechanism operating at the small area, or 'contextual' level. Two papers have investigated the problems of administrative boundaries in the context of mental health outcomes and found stronger associations with neighbourhood deprivation when measured in spatially adapted areas [25,26].

Previous papers have largely used measures of small area deprivation derived from routine national administrative census data, such as the Townsend and Carstairs indices of deprivation which are widely used in the UK as valid and reliable measures at census ward level [27]. Their disadvantage in multilevel investigations of people and places is that as generic measures, these indices do not offer a specific underlying model for a hypothesised relation between area level exposures and health outcome [3]. As a result, evidence for an independent effect of place, as measured by these deprivation indices, on the mental health status of individuals is inconsistent [7,8,10]. Other studies from Great Britain, the Netherlands and the USA have used other administrative sources of data on small area deprivation [9,11-13,17,18], household income [14-17] and mortality [16], but also with no strong evidence for a contextual effect on individual mental health.

Researchers have therefore argued for more specific measures of neighbourhood to provide more accurate underlying and testable models of how features of the neighbourhood could be hypothesised to influence the health status of individuals over and above the influences of personal risk factors [3]. A potentially useful but little investigated source of data on more specific aspects of the local area is the wide range of data on levels of benefits claimants held by the Department of Work and Pensions (DWP) at UK census ward level. Although it is possible that benefits data simply capture the socioeconomic dimension in a more accurate way than with measures like the Townsend index, it is theorised that they offer a more direct measurement of a wide range of local neighbourhood conditions and they have been shown in previous research to be useful for local joint planning processes to describe spatial variation in health and social needs at small area level [28,29]. These data may offer a more specific measure of neighbourhood conditions that can be hypothesised to affect health status as a first stage in developing testable models of relations between people, places and health.

In a previous multilevel analysis of population survey data from Caerphilly county borough, one of the 22 local government unitary authorities in Wales, UK, we found evidence for substantial contextual effects of ward-level economic inactivity on individual mental health [30]. These associations were stronger for individuals who were unavailable for work due to permanent sickness or disability. We wished to test these findings and extend the analysis using data from a questionnaire survey with complete coverage of the population of Wales. The study area of Wales is a country of 2.9 million people (2001 Census), a land of substantial socio-economic contrasts, from the post-industrialised south Wales valleys, to the major urban centres in Cardiff, Swansea, Newport and Wrexham, and with a large, sparsely populated rural interior. We derived further census ward-level or neighbourhood measures from DWP benefits data to investigate the hypotheses that (1) the mental health status of individuals is associated with contextual measures of low income, unemployment in the economically active (seeking work) and inactive (not available for work), and disability requiring daily care, after adjusting for personal risk factors for poor mental health, and (2) the associations between mental health and the contextual measures vary significantly between population sub-groups characterised by age group, gender, socioeconomic and employment status. We also compared the effect of the contextual benefits measures with the Townsend area deprivation score.

## Methods

### Sources of data

We used data on individuals from the Welsh Health Survey 1998, a postal questionnaire cross-sectional survey of one in 45 of the Welsh population aged between 17 and 104 years [31,32]. Local Research Ethics Committee approval was not required for secondary analyses of this dataset. 29,874 valid responses were returned, representing an adjusted response of 61%. Data items included age, gender, occupational status, social class, marital status, whether a carer, housing tenure and the Short Form (SF-36) health status questionnaire [33]. From the responses to the questions on occupation we derived three categories of employment status: (1) employed, (2) unemployed but economically active (seeking work) and (3) economically inactive (not available for work – student, retired, permanently sick or disabled, at home, other).

In Wales there were 908 wards defined in the 1991 census. The 1998 boundary revision re-defined these ward boundaries into 865 electoral divisions, mean population 3500, nested within the 22 unitary authority areas of local government. In this paper we retain the use of the term 'ward' rather than 'electoral division', for simplicity. Survey responses were obtained from individuals living in 833 (96.3%) of the 865 wards and from all 22 unitary authorities. The mean number of respondents per ward was 36 (min 1, max 195, inter-quartile range 18, 45). Of the 833 wards, 210 (25%) had less than 15 respondents.

### Mental health outcome measure

The mental health outcome measures available in the dataset were the Mental Health Inventory (MHI-5) and the mental component summary score (MCS) of the SF-36 [33]. We used the MHI-5 scale because the validity and reliability of the MHI-5 is well established [34] and the MCS had a greater proportion of missing data. Using the standard algorithm [33], the raw MHI-5 score was transformed by the data suppliers to a score taking values between zero to 100. On this scale, lower scores indicate worse mental health status. In this dataset, data on age were missing from 704 (2.4%) respondents. As in our previous analysis of this dataset, we restricted the analysis to respondents aged less than 75 years [10]. Of the remaining 26,175 respondents aged between 17 and 74 years, the mental health score was missing in 1200 (5.6%), so that the dataset for analysis included 24,975 individuals with a mean individual mental health score of 72 (standard deviation 19). The mean number of respondents per ward was 30, range 1 to 157.

### DWP benefits data

After the necessary permissions were obtained through collaboration with the Local Government Data Unit – Wales [35], a benefits data extract was received from the DWP for August 2001. Benefits fall into two main categories of means tested and non-means tested. Full details of each benefit are available from the DWP website [36]. The means tested benefits data made available for this study were Income Support (IS) and Income-based Job Seekers Allowance (IBJSA). Income Support is paid to people aged 16 to 59 years who are on low incomes and not working or work less than 16 hours a week, and have assets of less than ₤8,000 excluding their home. Income Support can be paid in addition to other benefits, such as Incapacity Benefit. Income-based Job Seekers Allowance replaced unemployment benefit and income support for unemployed people in 1996. It is payable to people aged from 16 years to pension age (currently 60 years for women and 65 years for men) who are also on low income and available for and actively seeking work. It is therefore a measure of unemployment in the economically active.

The non-means tested benefits data made available were: Incapacity Benefit and Severe Disablement Allowance; and Disability Living Allowance and Attendance Allowance. Incapacity Benefit and Severe Disablement Allowance are a measure of unavailability for work due to permanent sickness or disability, and thus a measure of economic inactivity. Incapacity Benefit is payable after the 28 weeks covered by Statutory Sick Pay to people aged between 16 years and pension age who have paid sufficient National Insurance contributions to qualify. The long-term rate of Incapacity Benefit is paid to those who have been sick for more than a year. Severe Disablement Allowance is claimed by people who have never been able to work, or who do not satisfy the conditions for Incapacity Benefit. New claims of Severe Disablement Allowance were withdrawn from April 2001, but existing claims are still paid.

Disability Living Allowance and Attendance Allowance are both measures of disability in people that require care. Disability Living Allowance is paid to people over the age of three months who are disabled and need help with personal care, mobility, or both. Payments are divided into care and mobility components and there are higher and lower rates of benefit depending on the level of assistance needed. Attendance Allowance is a benefit for people aged 65 years and over who need help with personal care or supervision, either during the day or at night.

### Calculation of ward-level contextual variables

The data were provided as the numbers of claimants in five-year age bands by ward and we calculated indirect age-standardised ward ratios standardised to the Caerphilly borough population. The population denominator for the quarter ending July 2001 was extracted from the General Practitioner age-sex register, the '*Exeter*' database, held by the former Gwent Health Authority and the Income-based Job Seekers Allowance ward ratio was calculated using the economically active denominator from the 2001 Census. Since Incapacity Benefit and Severe Disablement Allowance share the same underlying population at risk, we derived a new variable (IB/SDA) by aggregating the number of claimants of each benefit. With very small numbers over the age of 60, we used data on claimants and the total working age population denominator for people aged 16–59 in this study. Because the Disability Living Allowance and Attendance Allowance have an overlapping age range, we calculated the standardised ward ratio for the Disability Living Allowance and Attendance Allowance combined (DLA/AA), for all ages. We have previously calculated the Townsend score for this dataset using 1991 Census data on unemployment, owner occupation, car ownership and overcrowding [10].

### Statistical analysis

#### Descriptive statistics and uni-variable associations

We have previously published data on the individual-level variables and their univariable associations with the mental health score in a multilevel analysis of economic inactivity and social deprivation using the same dataset [10]. We calculated the descriptive statistics for the ward mean mental health score and the contextual variables, and the rank correlation matrix for the linear associations between them.

#### Multilevel modelling

The techniques of multilevel modelling have been well described [37-39]. We followed a similar modelling strategy to our previous analysis of the dataset [10] and specified a three-level multilevel analysis with 24,975 individuals at level 1, the 833 wards at level 2 and the 22 unitary authorities at level 3. Although the focus of interest in this paper was the fixed parameter estimates of the contextual variables rather than a detailed investigation of random variation at each spatial level in the model, the multilevel approach is still the appropriate analytical technique as it provides less biased fixed effect parameter estimates and gives appropriate standard errors when modelling a hierarchical dataset [37].

In the initial 'null' three-level model of random intercepts, the variation in the mental health score was modelled by random intercept terms for wards and unitary authorities and an individual random error term. In model A we fitted main effects for the same individual-level variables that were significantly associated with the mental health score as in the previous analysis [10]. In brief, we modelled age (centred on the mean value) and age centred squared (since the relation between age and mental health score was best approximated by a quadratic function). We modelled gender, social class (I& II, III non-manual, III manual, IV, V), married (yes/no), carer (yes/no), employment status (employed, unemployed economically active, unemployed economically inactive) and housing tenure (owner occupier, yes/no) as categorical variables, with missing data for each categorical variable included as a dummy term. As in our previous analysis, we included significant interaction terms between age and gender, and age and economic inactivity that improved the fit of the model to the data [10].

Models B and C investigated contextual effects. To address the first hypothesis of the study, a separate model B was fitted for each benefits data variable and the Townsend score in turn by entering these variables one by one to the compositional model A. Each variable was modelled as a z-score, obtained by subtracting the mean and dividing by the standard deviation of the distribution of scores (Table 1). This had two advantages over modelling the raw scores: firstly, the magnitude of the z-parameter estimates represented the change in predicted mental health score for a change in magnitude of the contextual variable by 1 standard deviation; and secondly, the magnitude of the parameter estimates for the benefits data could be directly compared between models. We investigated the possibility of non-linear effects by modelling quadratic terms for the contextual variables. We then ran a further four models including the Townsend score with each benefit measure in turn to assess whether any contextual effects might be shared between the two measures. We also investigated the second hypothesis of the study in model C. Here we assessed whether any associations between mental health and the contextual data varied with population sub group by modelling cross-level interactions between the ward-level data and age group in ten-year bands, gender, social class and employment status of individuals. The age group models included age group as a categorical variable to estimate the fixed effects – all other models included age and age squared as continuous variables.

**Table 1:** Descriptive statistics for the number and percent of benefits claimants for census wards in Wales, August 2001

	**Mean**	**Median**	**SD**	**Min.**	**Max.**	**IQR**	
						**25**	**75**
**Numbers of claimants**							
IS	271.6	168.0	287.9	16	2390	96	346
IBJSA	44.0	28.0	50.4	0	419	15	54
IB/SDA	255.4	159.0	240.6	16	1634	93	336
DLA/AA	345.0	237.0	295.4	34	1910	141	460
							
**Percent claimants**							
IS	9.0	8.3	4.7	0.6	51.1	5.6	11.4
IBJSA	1.9	1.7	1.2	0	9.4	1.0	2.5
IB/SDA	11.0	10.0	5.3	1.3	43.6	7.1	13.9
DLA/AA	9.8	9.0	4.2	1.4	39.8	6.8	12.1

IS: Income Support

IBJSA: Income-based Job Seekers Allowance

IB/SDA: Incapacity Benefit, Severe Disablement Allowance

DLA/AA: Disability Living Allowance and Attendance Allowance

The models were estimated using the iterative generalised least squares method in MLwiN software followed by Markov Chain MonteCarlo (MCMC) estimation [39]. Model fitting was assessed using the deviance statistic. The difference in this between two models follows a chi-square distribution with the number of degrees of freedom equal to the difference in the numbers of parameters between the models [37]. 95% credible interval estimates for the mean intercept and the random variances at individual, ward and unitary authority levels were derived from the MCMC analysis. The validity of the final model was assessed using standard diagnostic plots of residuals at each level in the model. Although the MHI-5 scale is negatively skewed, our previous analysis of this dataset has shown robustness to the standard regression assumptions of Normality [10].

## Results

### Descriptive statistics and univariable analyses

Table 1 shows the descriptive statistics for the number and percent of claimants for the four benefits for wards in Wales. The number and percent of claimants was smaller for the Income-based Job Seekers Allowance than the other three benefits, with a mean claimant rate of 1.9%, and one ward had no recorded claimants. These claimant levels are similar to England (2.0%) and Scotland (2.3%), based on comparative data available for May 2002 [40]. The percent claimants for Income support was 9.0%, compared to 8.2% in England and 10.2% in Scotland in May 2002 [41]. The percent claimants for Incapacity Benefit and Severe Disablement Allowance combined was 11.0%, compared to 6.9% in England and 10.3% in Scotland in February 2003 [42]. The percent claimants for Disability Living Allowance and Attendance Allowance combined was 9.8%, compared to 6.2% in England and 8.0% in Scotland in February 2003 [43,44].

Table 2 shows the descriptive statistics for the ward mean mental health scores and the contextual covariates. The distributions of the indirectly age-standardised ratios are all positively skewed. The ecological rank correlation coefficients between the ward mean mental health score and the contextual covariates are shown in Table 3. The mean mental health score was significantly and negatively correlated with each contextual variable. Wards with higher levels of benefit claimants were associated with lower mean mental health scores. Although the magnitudes of the correlations were moderate, the strength of the correlations was higher for the incapacity and disability benefits.

**Table 2:** Descriptive statistics for the ward mean mental health score, Townsend score and benefits data^a ^(n = 833)

	**Mean**	**Median**	**SD**	**Min.**	**Max.**	**IQR**	
						**25**	**75**
1. Mean mental health score	72.5	72.7	5.2	53.7	88.6	69.2	76.3
2. Townsend score	-0.02	-0.2	3.3	-7.4	12.2	-2.4	2.0
3. IS	91.8	85.7	45.3	7.2	280.9	58.6	117.1
4. IBJSA	95.9	87.0	55.0	0.0	426.1	55.3	127.9
5. IB/SDA	92.9	82.8	46.2	20.6	246.0	57.3	121.9
6. DLA/AA	94.6	87.6	36.6	27.1	230.5	65.5	120.1

a: modelled as an indirectly age-standardised ratio where all-Wales = 100

IS: Income Support

IBJSA: Income-based Job Seekers Allowance

IB/SDA: Incapacity Benefit, Severe Disablement Allowance

DLA/AA: Disability Living Allowance and Attendance Allowance

**Table 3:** Spearman's rank correlation coefficients (95% CIs) for electoral division mean mental health score and contextual covariates^a^

	**1**	**2**	**3**	**4**	**5**	**6**
1. Mean mental health score	1	-0.143 -0.209, -0.076	-0.183 -0.248, -0.117	-0.154 -0.220, -0.087	-0.227 -0.290, -0.162	-0.228 -0.291, -0.163
2. Townsend score		1	0.870 0.852, 0.886	0.701 0.665, 0.734	0.742 0.710, 0.771	0.705 0.669, 0.738
3. IS			1	0.708 0.672, 0.740	0.898 0.884, 0.910	0.871 0.854, 0.886
4. IBJSA				1	0.625 0.582, 0.665	0.543 0.493, 0.589
5. IB/SDA					1	0.931 0.921, 0.940
6. DLA/AA						1

a: modelled as a z-score

All correlations p < 0.01

IS: Income Support

IBJSA: Income-based Job Seekers Allowance

IB/SDA: Incapacity Benefit, Severe Disablement Allowance

DLA/AA: Disability Living Allowance and Attendance Allowance

### Multilevel models

#### Random intercepts null model

The null model found that 97.0% of the random variation in the mental health score occurred at the individual level, with 1.3% at the ward and 1.7% at the unitary authority level (Table 4). The mean intercept mental health score was 72.06 and Table 4 shows the intercepts for the 833 wards varied around this mean intercept with a variance of 4.59 (standard error, (SE) 0.86, credible interval 3.05, 6.31).

**Table 4:** Variance components for three-level multilevel mental health models

**Model**	**Level 1: Individual**		**Level 2: Ward**		**Level 3: Unitary authority**	
	
	**Variance (SE)**	**2.5^th^- 97.5^th ^credible estimates**	**Variance (SE)**	**2.5^th^- 97.5^th ^credible estimates**	**Variance (SE)**	**2.5^th^- 97.5^th ^credible estimates**
**Null model**	352.1 (3.21)	345.9, 358.5	4.59 (0.86)	3.05, 6.31	6.24 (2.33)	3.09, 12.08
% of total random variance	97.0	95.4, 98.0	1.27	0.84, 1.74	1.72	0.86, 3.27
**Model A (Null + individual variables^a^)**	335.7 (3.05)	329.8, 341.8	1.72 (0.61)	0.60, 2.98	5.22 (1.96)	2.58, 10.27
% of total random variance	98.0	96.6, 98.8	0.50	0.17, 0.87	1.52	0.76, 2.95
**Model B (model A + contextual variables)**						
**Townsend score**	324.8 (2.95)	319.1, 330.7	0.52 (0.31)	0.14, 1.33	3.14 (1.20)	1.51, 6.12
% of total random variance	98.9	98.1, 99.5	0.02	0.00, 0.17	0.94	0.45, 1.84
**Income support**	324.8 (2.94)	319.1, 330.7	0.27 (0.25)	0.04, 0.98	2.69 (1.04)	1.27, 5.29
% of total random variance	99.2	98.4, 99.6	0.01	0.00, 0.12	0.80	0.38, 1.60
**IBJSA**	324.8 (2.95)	319.1, 330.7	0.58 (0.34)	0.17, 1.42	4.57 (1.69)	2.28, 8.78
% of total random variance	98.6	97.3, 99.3	0.02	0.00, 0.20	1.37	0.69, 2.61
**IB/SDA**	324.6 (2.94)	318.9, 330.5	0.27 (0.25)	0.04, 0.97	1.45 (0.61)	0.61, 2.92
% of total random variance	99.6	99.1, 99.8	0.01	0.00, 0.12	0.44	0.00, 0.91
**DLA/AA**	324.7 (2.94)	319.0, 330.6	0.34 (0.27)	0.07, 1.07	1.26 (0.55)	0.51, 2.61
% of total random variance	99.6	99.2, 99.8	0.04	0.00, 0.20	0.38	0.15, 0.80

a The individual-level variables were age, age squared, gender, social class, marital status, carer status, employment status, and housing tenure.

IS: Income Support

IBJSA: Income-based Job Seekers Allowance

IB/SDA: Incapacity Benefit, Severe Disablement Allowance

DLA/AA: Disability Living Allowance and Attendance Allowance

#### Model A: compositional effects

Addition of the compositional fixed effects to the null model to estimate model A resulted in reductions in the higher level random variances, to 0.5 % at ward and 1.5% at unitary authority level (Table 4). The relation between mental health and age was best modelled by a quadratic function so that lower mental health scores were found in middle age, compared to younger or older age groups. Lower mental health scores were associated with female gender, lower social class, not being married, being a carer, unemployed (both economically active and inactive) and living in non-owner occupied housing. Including interactions between the linear and quadratic terms for age and gender, and age and unemployment significantly improved the fit of model A [10].

#### Model B: contextual effects

Table 4 shows the substantive reductions in the area-level random variances in the respective contextual models, with greater reductions at the ward-level. In model B, the Townsend score and the benefits variables were all significantly and negatively associated with the mental health score of individuals, after adjusting for individual risk factors for poor mental health (Table 5). Thus, living in a ward with high levels of deprivation or claimants was associated with poorer mental health. Since each variable was modelled as a z-score, the parameter estimate is the effect size. The estimates are negative and so the effect size is interpreted as a decrease in the predicted mental health score for an increase in magnitude of the contextual variable by 1 standard deviation An effect size of 0.2 is generally accepted to be small, 0.5 moderate and 0.8 large, so that the effects of these benefits data are substantial [45]. The effect sizes were similar for the Townsend score and the two income-based means tested benefits at around -1.1 (SE 0.13), and substantially higher for the two disability based non-means tested benefits at around -1.6 (SE 0.15). The estimates for the benefits variables remained significant after inclusion of the Townsend score in the respective models, with the exception of income-based job seekers allowance in which both variables were significant.

**Table 5:** Parameter estimates (SE) for benefits data in mental health models

**Variable**	**Parameter estimate**^a^	**SE**	**95% CI**	**Deviance**
Townsend score	-1.072	0.128	-1.314, -0.819	215301.8
IS	-1.187	0.125	-1.423, -0.941	215305.0
IBJSA	-1.073	0.143	-1.346, -0.786	215305.3
IB/SDA	-1.652	0.147	-1.932, -1.362	215285.9
DLA/AA	-1.656	0.155	-1.950, -1.349	215294.7
Townsend + IS	-0.147 -1.057	0.251 0.248	-0.630, 0.341 -1.541, -0.583	215308.2
Townsend + IBJSA	-0.800 -0.416	0.182 0.204	-0.808, -0.011 -1.163, -0.447	215309.2
Townsend + IB/SDA	-0.062 -1.591	0.189 0.222	-0.432, 0.303 -2.024, -1.159	215289.5
Townsend + DLA/AA	-0.261 -1.417	0.177 0.219	-0.610, 0.082 -1.846, -0.987	215299.2

a Adjusted for age, age squared, gender, social class, marital status, carer status, employment status, and housing tenure.

IS: Income Support

IBJSA: Income-based Job Seekers Allowance

IB/SDA: Incapacity Benefit, Severe Disablement Allowance

DLA/AA: Disability Living Allowance and Attendance Allowance

In view of the positive skew of the distributions of the Townsend score and the benefits variables we checked for linearity by deriving Loess plots using a non-parametric smoothing method [46]. This showed no signs of non-linearity. We did repeat the analysis by modelling these variables as four-level categorical variables using the 25^th^, median, and 75^th ^centile values as cut-points (data shown in Table 2). We found the same pattern of significant associations with a gradient across the categories, suggesting that the results of modelling the contextual variables as continuous variables were not sensitive to outlier effects (Table 6).

**Table 6:** Parameter estimates (SE) for the contextual variables modelled as categorical variables

**Variable**	**Parameter estimate**^a^	**SE**	**95% CI**
**Townsend score**			
Least deprived (reference)			
2	-0.782	0.373	-1.512, -0.054
3	-1.420	0.370	-2.146, -0.681
Most deprived	-2.320	0.367	-3.035, -1.585
**IS**			
Lowest claimants (reference)			
2	-0.413	0.363	-1.114, 0.293
3	-1.558	0.368	-2.288, -0.835
Highest claimants	-2.859	0.368	-3.570, -2.130
**IBJSA**			
Lowest claimants (reference)			
2	-1.442	0.366	-2.154, -0.728
3	-1.518	0.373	-2.275, -0.778
Highest claimants	-2.749	0.383	-3.494, -1.959
**IB/SDA**			
Lowest claimants (reference)			
2	-0.943	0.376	-1.672, -0.200
3	-1.921	0.378	-2.651, -1.169
Highest claimants	-3.749	0.417	-4.555, -2.913
**DLA/AA**			
Lowest claimants (reference)			
2	-0.564	0.378	-1.297, -1.182
3	-1.928	0.384	-2.673, -1.162
Highest claimants	-3.858	0.433	-4.699, -2.998

a Adjusted for age, age squared, gender, social class, marital status, carer status, employment status, and housing tenure.

IS: Income Support

IBJSA: Income-based Job Seekers Allowance

IB/SDA: Incapacity Benefit, Severe Disablement Allowance

DLA/AA: Disability Living Allowance and Attendance Allowance

#### Model C: cross-level interactions

**Figure 1:**
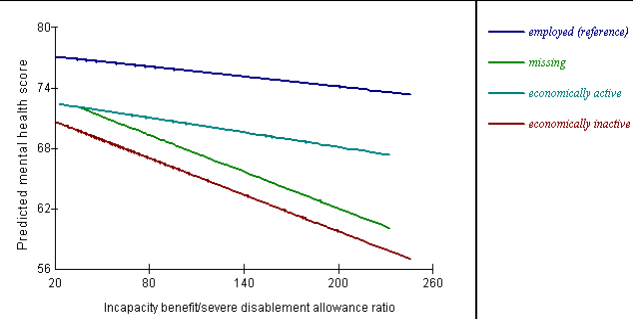
Cross-level interaction between Incapacity Benefit/Severe Disablement Allowance ratio and individual employment status.

**Figure 2:**
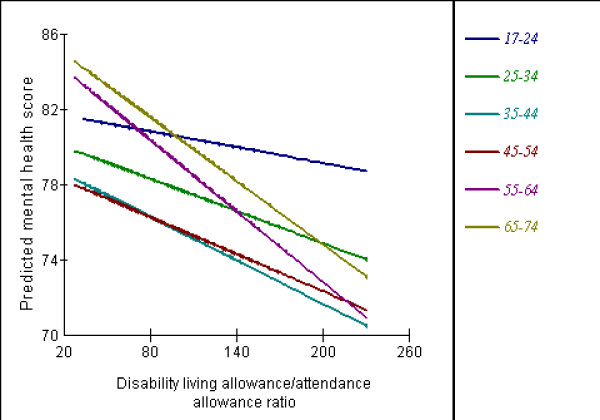
Cross-level interaction between Disability Living Allowance/Attendance Allowance ratio and age group.

Tables 7 and 8 show the parameter estimates (SE) for the cross-level interactions between the contextual variables and the categories of age, gender, social class and employment status. The consistent finding for all of the contextual variables modelled was the significant cross-level interactions with the economically inactive category of employment status. Thus the significant gradients of association of the contextual variables with individual mental health status were more steeply negative for individuals who reported being economically inactive compared to the other categories of employment status. These cross-level interactions are illustrated for the Incapacity Benefit and Severe Disablement Allowance variable in Figure 1. The effect of the Townsend score and the income-based means tested benefits did not vary with age group, but the effects of the Incapacity Benefit and Severe Disablement Allowance and the Disability Living Allowance and Attendance Allowance variables were stronger in the over 55 years age groups compared to the younger age groups. Thus the effect on mental health of living in an area of high economic inactivity and disability is greater for older age groups (illustrated in Figure 2). The importance of these significant interactions is shown by the magnitude of the effect sizes which are substantively larger than their main effects (Tables 7 &8). The effect of the contextual variables did not vary significantly with gender or social class, except for the 'other/missing' category of social class which mainly includes the economically inactive who are not coded into one of the formal categories.

**Table 7:** Parameter estimates (SE) for cross-level interactions for the Townsend score, Income Support and Income-based Jobseekers Allowance

		**Townsend score**		**IS**		**IBJSA**	
**Variable**	**Category**	**Parameter estimate^a ^(SE)**	**95% CI**	**Parameter estimate^a ^(SE)**	**95% CI**	**Parameter estimate^a ^(SE)**	**95% CI**

Age group^b ^(17–24 reference)	Main effect of contextual variable	-0.751 0.335	-1.411, -0.095	-0.558 0.334	-1.210, 0.090	-0.603 0.366	-1.328, 0.109
	25–34	-0.129 0.426	-0.968, 0.711	-0.386 0.422	-1.188, 0.442	-0.351 0.458	-1.236, 0.551
	35–44	0.012 0.419	-0.804, 0.828	-0.243 0.416	-1.041, 0.569	-0.187 0.455	-1.069, 0.695
	45–54	-0.244 0.413	-1.067, 0.558	-0.511 0.411	-1.327, 0.293	-0.373 0.443	-1.234, 0.497
	55–64	-0.873 0.425	-1.685, -0.011	-1.383 0.423	-2.182, -0.534	-0.819 0.459	-1.695, 0.109
	65–74	-0.766 0.443	-1.633, 0.108	-1.122 0.436	-1.987, -0.279	-0.968 0.477	-1.901, -0.037
Gender (male reference)	Main effect of contextual variable	-0.830 0.175	-1.174, -0.487	-0.950 0.173	-1.282, -0.614	-0.747 0.194	-1.132, -0.364
	Female	-0.428 0.224	-0.879, 0.005	-0.415 0.219	-0.856, 0.008	-0.573 0.240	-1.059, -0.107
Social class (I&II reference)	Main effect of contextual variable	-0.899 0.233	-1.354, -0.435	-1.028 0.242	-1.506, -0.545	-1.191 0.261	-1.696, -0.671
	III non-manual	-0.272 0.320	-0.908, 0.349	-0.212 0.324	-0.844, 0.421	0.083 0.352	-0.599, 0.771
	III manual	0.317 0.361	-0.398, 1.026	0.282 0.358	-0.428, 0.982	0.617 0.390	-0.157, 1.379
	IV	-0.237 0.352	-0.922, 0.476	-0.178 0.348	-0.855, 0.527	0.199 0.374	-0.533, 0.958
	V	0.454 0.600	-0.731, 1.602	0.118 0.562	-0.983, 1.189	0.438 0.606	-0.762, 1.613
	Other/missing	-1.168 0.400	-1.949, -0.391	-1.058 0.393	-1.829, -0.277	-0.487 0.418	-1.299, 0.340
Employment Status (employed reference)	Main effect of contextual variable	-0.460 0.162	-0.777, -0.136	-0.409 0.160	-0.723, -0.084	-0.534 0.181	-0.890, -0.166
	Economically active	0.897 0.685	-0.452, 2.247	0.570 0.652	-0.713, 1.860	0.021 0.677	-1.310, 1.356
	Economically inactive	-1.548 0.234	-2.006, -1.090	-1.867 0.228	-2.313, -1.423	-1.353 0.251	-1.848, -0.866
	Missing	-0.164 1.625	-3.303, 2.903	-0.180 1.464	-3.001, 2.607	2.505 1.476	-0.375, 5.352

a Adjusted for age, age squared, gender, social class, marital status, carer status, employment status, and housing tenure.

b Only the agegroup model has agegroup main effects, all others model age and age squared

IS: Income Support

IBJSA: Income-based Job Seekers Allowance

IB/SDA: Incapacity Benefit, Severe Disablement Allowance

DLA/AA: Disability Living Allowance and Attendance Allowance

**Table 8:** Parameter estimates (SE) for cross-level interactions for the Incapacity benefit/Severe Disablement Allowance/Attendance Allowance

		**IB/SDA**		**DLA/AA**	
**Variable**	**Category**	**Parameter estimate^a ^(SE)**	**95% CI**	**Parameter estimate^a ^(SE)**	**95% CI**

Age group^b ^(17–24 reference)	Main effect of contextual variable	-0.523 0.349	-1.215, 0.158	-0.722 0.362	-1.427, 0.000
	25–34	-0.677 0.436	-1.515, 0.177	-0.396 0.464	-1.307, 0.528
	35–44	-0.981 0.429	-1.806, -0.142	-0.718 0.448	-1.604, 0.153
	45–54	-0.992 0.420	-1.825, -0.169	-0.590 0.433	-1.418, 0.271
	55–64	-2.010 0.432	-2.828, -1.141	-1.931 0.454	-2.848, -1.035
	65–74	-1.789 0.444	-2.669, -0.927	-1.843 0.466	-2.767, -0.946
Gender (male reference)	Main effect of contextual variable	-1.468 0.188	-1.842, -1.109	-1.453 0.196	-1.842, -1.079
	Female	-0.325 0.224	-0.768, 0.111	-0.363 0.233	-0.823, 0.089
Social class (I&II reference)	Main effect of contextual variable	-1.460 0.256	-1.961, -0.948	-1.303 0.264	-1.815, -0.776
	III non-manual	-0.071 0.338	-0.740, 0.592	-0.230 0.351	-0.927, 0.457
	III manual	0.097 0.362	-0.605, 0.796	-0.080 0.377	-0.814, 0.651
	IV	-0.396 0.356	-1.086, 0.289	-0.678 0.369	-1.391, 0.032
	V	0.108 0.583	-1.045, 1.232	-0.239 0.605	-1.430, 0.929
	Other/missing	-1.017 0.403	-1.810, -0.226	-1.217 0.419	-2.045, -0.395
Employment Status (employed reference)	Main effect of contextual variable	-0.742 0.181	-1.107, -0.390	-0.684 0.189	-1.604, 0.313
	Economically active	-0.342 0.700	-1.723, 1.040	-0.509 0.752	-1.994, 0.975
	Economically inactive	-2.042 0.231	-2.485, -1.599	-2.183 0.240	-2.644, -1.721
	Missing	-2.079 1.741	-5.503, 1.232	-2.587 1.909	-6.343, 1.049

a Adjusted for age, age squared, gender, social class, marital status, carer status, employment status, and housing tenure.

b Only the agegroup model has agegroup main effects, all others model age and age squared

IS: Income Support

IBJSA: Income-based Job Seekers Allowance

IB/SDA: Incapacity Benefit, Severe Disablement Allowance

DLA/AA: Disability Living Allowance and Attendance Allowance

### Model validity

Standard diagnostic plots available in MLwiN showed that the individual, ward and unitary authority level residuals in each contextual model were independent of the explanatory variables and the predicted mental health scores. The Spearman rank correlation coefficients for the linear associations between the ward standardised residual and the contextual variable were not significantly different from zero in each case. Re-running the models excluding wards with less than 15 respondents (now including 22,740 cases) found little difference in the parameter estimates.

## Discussion

We have used both means tested and non-means tested benefits data from the Department of Work and Pensions to quantify characteristics of the neighbourhood for an analysis of people, places and mental health. Higher levels of claimants of Income Support, Income-based Job Seekers Allowance, Incapacity Benefit and Severe Disablement Allowance, and Disability Living Allowance and Attendance Allowance, modelled at ward-level were significantly associated with poorer individual mental health status after adjusting for the characteristics of individuals associated with poor mental health. All of these contextual associations had large effect sizes. The income-based means tested benefits were similar in the size of their effect to the Townsend score, reflecting their proxy measurement of material and social deprivation. The non-means tested benefits data that were proxy measures of disability – unavailability for work from permanent sickness and disability requiring care – showed substantively stronger contextual effects on individual mental health than the means tested benefits, suggesting that these measures may be capturing a different dimension to socio-economic deprivation. We found further evidence to support this since inclusion of both the Townsend score and each non-means tested benefit in the models made little difference to the size of the disability effects and the Townsend score was no longer significant. The area-level random variances were small in comparison to the random variation at individual level, but comparable to other multilevel analyses of mental health outcomes [7-18]. Small area-level random variances are entirely compatible with large estimates for the area-level fixed effects and do not imply an absence of the importance of area [47,48]. The smallest variances were generally shown for the disability based non-means tested benefits, and this is consistent with their explaining a greater proportion of the variation in mental health scores than the Townsend score and the means tested benefits.

We found that the strength of the contextual effect on mental health for all the modelled variables was significantly stronger in people who were economically inactive from permanent sickness or disability. The contextual effect of the disability benefits was stronger also in the older age groups. No differences in contextual effects were found for gender and social class, with the exception of the 'other' category of social class, which included people who had never worked as a result of disability and were therefore economically inactive. These findings are interesting as they give insights into a possible causal pathway. We have previously shown that neighbourhood social cohesion may be an effect modifier of associations between area income deprivation and individual mental health [21]. It is plausible that neighbourhoods with high rates of people with disabilities will have lower levels of social cohesion, due to the difficulties of social engagement resulting from chronic and disabling illness, and this effect is likely to be stronger in older age groups.

### Strengths and weaknesses

The major strength of the study is that the Welsh Health Survey contains detailed and representative information on around 1 in 80 of the Welsh resident population aged 18 to 74, resident in 833 of the 865 wards. This high sampling fraction offers a different analytical perspective to other studies which analyse datasets which cover Great Britain but with a very small sampling fraction of both individuals and areas [7,8,12,13]. A second strength is that we have investigated the wider use of DWP benefits data from the total population of Wales as contextual measures of income deprivation, work deprivation in the economically active and inactive, and disability requiring assistance with personal care.

The main weaknesses of the study are inherent in all cross-sectional epidemiological analyses [49]. We cannot exclude the possibility of a health selection effect, in which people move into poorer neighbourhoods as a result of poor health and thereby bias the observed associations between neighbourhood attributes and health outcome. However, the bias could be in either direction [50] and a longitudinal study is required to assess causality. We cannot conclude that we have found strong evidence for contextual effects in the absence of testing hypothesised causal pathways in a longitudinal analysis. We have used the administratively defined ward as a proxy for neighbourhood. However, the effect of non-differential misclassification of individuals into an inappropriate administrative boundary is to bias associations with the area exposure to the null and so this would not explain our results [51]. Lower response rates from some subgroups are an unavoidable feature of population surveys [52]. Because this study is investigating the relationship between variables, rather than making inferences about population prevalence, non-response bias could be in either direction if the relationships between the variables are substantially different in those subgroups from the rest of the population.

The benefits data have some drawbacks. Only person-based data were available so it was not possible to carry out a separate analysis to investigate gender effects. Small numbers of claimants in some wards resulted in the possibility of influential effects from outlying values. However, the results were substantively the same after analysing the benefits data as categorical variables, suggesting that outlier values did not adversely affect the results. Variation in the uptake of benefits might be related to social and geographical factors rather than need [28,29] and these so-called 'supply-side effects' could bias contextual effects away from the null if lower uptake was a feature of less socio-economically deprived neighbourhoods. One of the limitations of the incapacity benefit data is that there is evidence that claiming incapacity benefits may have been a response to the poor socio-economic conditions resulting from the loss of jobs in the traditional coal and steel industries in the south Wales valleys in the 1980s [53]. Thus post-industrialised wards in parts of Wales may have higher level of claimants than expected for their levels of morbidity. This could bias the results towards significant effects of contextual economic inactivity, but we do not have any data to investigate this further. A further limitation of the data is that it was not possible to disaggregate claims data by diagnostic category, so that we could not model a ward-level measure of incapacity benefit for claimants with poor mental health. Temporal mismatch between the Townsend score (1991), survey data (1998) and benefits data (2001) is a further limitation, arising from constraints of data availability.

### Comparison to previous literature

We have been unable to find any previous studies that use benefits data to model neighbourhood context and health outcome. Data on benefits claimants have been used at ward level in the four UK countries as part of a wide range of data used in the construction of four different multidimensional deprivation indices [54-57]. One multilevel study of places, people, and mental functional health set in East Anglia, UK, used the 2000 Index of Multiple Deprivation in England as a measure of ward deprivation [9]. This index is aggregated from six domains of deprivation, namely: income; employment; health and disability; education, skills and training; housing; and geographical access to services [54]. Benefits data are included in the construction of the income, employment, and health and disability domains, but it is likely that any specific effect is lost within the large degree of aggregation involved in creating a single index from a wide range of variables, and the paper reported only weak evidence for an association between area deprivation and individual mental functional health. A previous multilevel analysis of data from the Welsh Health Survey used the Welsh Index of Multiple Deprivation [55] to test the hypothesis that individuals living in more deprived areas would have worse mental health [11]. The study found that the index used as a summary measure of deprivation at the unitary authority level based on the median rank ward score was associated with poor mental health status. However, as in the study from England [9], the six domains of deprivation were not modelled separately and so more specific associations between aggregate benefits data and mental health could not be assessed.

## Conclusion

This study provides substantive evidence in support of contextual effects on mental health, and in particular the importance of economic inactivity from permanent sickness or disability at both contextual and individual level. Benefits data obtainable for small geographical areas from the DWP offer a more specific measure of neighbourhood characteristics than generic deprivation indices and offer a starting point to hypothesise possible causal pathways from neighbourhood context to individual mental health status. Benefits data are centrally collated on a monthly basis and therefore have the potential to measure changes in neighbourhood context over time for use in longitudinal studies that can assess causal pathways.

## Competing interests

The authors declare that they have no competing interests.

## Authors' contributions

DF designed the study, carried out the analysis, and wrote the manuscript. FD provided statistical advice. KL advised on the measurement of the mental health outcome. All authors critically revised the manuscript and read and approved the final version.

## Pre-publication history

The pre-publication history for this paper can be accessed here:


